# Can internet use promote farmers to adopt chemical fertilizer reduction and efficiency enhancement technology in China?—an empirical analysis based on endogenous switching probit model

**DOI:** 10.1371/journal.pone.0308300

**Published:** 2024-08-30

**Authors:** Zhifei Ma, Huan Huang, Xiangmin Zhang, Dongxue Qin, Xiaodi Li

**Affiliations:** Henan Key Laboratory for Synergistic Prevention of Water and Soil Environmental Pollution, School of Geographic Sciences, Xinyang Normal University, Xinyang, China; CDC Foundation, UNITED STATES OF AMERICA

## Abstract

This study examines the impact of internet usage on farmer’s adoption behavior of fertilizer reduction and efficiency enhancement technologies in China. Based on 1,295 questionnaires in Henan Province, this study constructs a counterfactual analysis framework and used endogenous switching probit model to analyze the effects and pathways of internet usage on farmer’s adoption behavior of chemical fertilizer reduction and efficiency enhancement technologies. The results indicate that. (1) The proportion of farmers adopting chemical fertilizer reduction and efficiency enhancement technologies is 60.15%, while the proportion of farmers not adopting these technologies is 39.85%. (2) Internet usage directly influences farmers’ adoption of fertilizer reduction and efficiency enhancement technologies. According to counterfactual assumption analysis, if farmers who currently use the Internet were to stop using it, the probability of them adopting these technologies would decrease by 28.09%. Conversely, for farmers who do not currently use the Internet, if they were to start using it, the probability of them adopting fertilizer reduction and efficiency enhancement technologies would increase by 40.67%. (3) Internet usage indirectly influences farmers’ adoption behavior through mediating pathways of expected benefits and risk perception. In addition, social networks negatively moderate the impact of internet usage on farmers’ behavior of chemical fertilizer reduction and efficiency enhancement technologies.

## Introduction

The United Nations’ 2030 Agenda for Sustainable Development emphasizes the goals of eradicating hunger, achieving food security, improving nutritional status, and promoting sustainable agricultural development. The application of fertilizers is an important means to achieve these goals, as experiences in both developed and developing countries have shown the significant impact of fertilizers on national food security production [1]. The use of fertilizers is increasingly recognized as a key factor in achieving increased food production and agricultural economic growth [2]. However, prolonged, excessive, and inefficient fertilizer usage leads to issues such as increased agricultural production costs, resource wastage, non-point source pollution in agriculture, degradation of land quality, and reduced agricultural product quality [3]. This dual constraint of environmental concerns and product quality presents hurdles to food production, creating a conundrum in the pursuit of food security development [4]. Consequently, there is a global consensus on the imperative need to explore methods for promoting the reduced and efficient utilization of fertilizers. Advancing technologies aimed at reducing and efficient fertilizer use stands out as a crucial measure in advancing global food security, fostering sustainable agricultural development, and facilitating the transition towards environmentally conscious practices [4,5].

China has perennially grappled with the conundrum of an imbalanced scenario characterized by a substantial population against the backdrop of limited resources [6]. Confronted with the exigencies of rapid population expansion, China has historically pursued a developmental paradigm emphasizing heightened grain output, notably through the intensive application of agricultural chemicals such as fertilizers and pesticides. This approach has yielded commendable success, effectively addressing the imperative of food security for China’s nearly 1.4 billion population [7]. However, in recent years, the contribution of chemical fertilizers to grain growth in China has declined from 30% to 40% in the 1980s to around 10% at present [8]. This decline can be attributed to the excessive use of chemical fertilizers, which has led to a series of negative impacts, such as agricultural non-point source pollution, degradation of soil ecosystems, and increased agricultural production inputs [9]. In the broader context of agricultural sustainable development, the Ministry of Agriculture introduced the ’Zero Growth Action Plan for Fertilizer Usage by 2020’ in 2015. This plan aims to promote the reduction and efficiency improvement of fertilizer application in agriculture. The data indicates that in 2017, China’s usage of agricultural fertilizers decreased by 2.71% compared to 2015, and the fertilizer utilization efficiency for major grain crops increased by nearly 4% [10]. This indicates that China has made positive progress in scientific fertilization work and has achieved the goal of zero growth in fertilizer usage ahead of schedule. However, although China has achieved the goal of zero growth in fertilizer usage and has controlled the growth rate, the total volume of fertilizer is still high [11].China’s total fertilizer usage amounts to 58.5941 million tons, accounting for 33% of the world’s total fertilizer consumption. The application rate per unit of arable land is 434.46 kilograms per hectare, approximately 3.9 times higher than the global average and greatly exceeding the internationally recognized safe limit of 225 kilograms per hectare for fertilizer application [12]. The 2019’s No.1 Central Document emphasized the need to continue the “Launching the Agricultural Fertilizer Conservation Campaign and achieve negative growth in fertilizer usage”. The “14th Five-Year Plan” also sets the goal of reducing the amount of fertilizer used in grain crops to a level that meets the “appropriate fertilization level” for sustainable agricultural development by 2025. Therefore, the effective realization of a reduction in fertilizer usage while concomitantly enhancing efficiency remains a pivotal and formidable challenge that government department and academic community concentrated attention to, and farmers, as the principal agents in achieving this objective, wield the pivotal role in resolving this issue. However, the prevalent low adoption willingness and implementation levels among farmers directly impact the progress and effectiveness of the agricultural green transformation [13].

Reducing fertilizer usage is key to sustainable agricultural development, and there have been many studies focusing on farmers’ fertilizer application behavior. On one hand, starting from the perspective of farmers as the main producers, existing research focuses on the impact of farmers’ individual characteristics and household features on their fertilizer application. Objective characteristics such as the age, gender, and educational level of the household head are taken into account. In addition, subjective characteristics such as risk aversion [14], fertilizer cognition [15] and soil fertility cognition [16], all have an impact on the reduction and efficiency enhancement technology of fertilizer consumption by farmers. Risk aversion not only increases the likelihood of farmers choosing excessive fertilizer application but also significantly intensifies the degree of excessive fertilizer use. Family characteristics include the degree of part-time employment [17], Family cultivated land area [18], Supply of family agricultural labor force [5], Social networks and other factors [19].In this regard, family agricultural labor and the level of diversification have a significant positive impact on reducing fertilizer input by farmers. The larger the family cultivated land area and the greater the supply of agricultural labor force, the more likely farmers are to reduce excessive use of fertilizers. At the same time, social networks not only reduce the likelihood of farmers choosing excessive use of fertilizers but also reduce the extent of excessive fertilizer application by farmers. On the other hand, starting from the agricultural production conditions, existing literature has focused on land scale [20], The number of cultivated land plots [21], Stability of agricultural land tenure [22], fragmentation level of land and other factors [23]. Farmland scale is divided into three categories: farmland management scale, plot scale, and contiguous scale. Among these, as the plot scale increases, the reduction in fertilizer application also increases, and as farmers expand contiguous farming, the reduction amount also increases. Enlarging the plot scale and expanding the management scale can promote the reduction of fertilizer application. Moreover, stable land tenure and long-term investments by farmers, such as the application of organic fertilizers and the cultivation of green manure, have incentive effects. The fragmented distribution of land can hinder the adoption of fertilizer-saving agricultural technologies and increase fertilizer input. If fragmented land can be consolidated into contiguous scales through a socialized approach to agricultural production, it can help control excessive fertilizer input.

In comparison, there is very limited empirical evidence regarding the impact of the internet on farmers’ reduction of fertilizer usage and efficiency improvement. In the context of rapid development of rural digital economy, traditional agricultural technology dissemination methods have disadvantages such as low efficiency, limited content, and poor timeliness compared to internet-based promotion methods [24]. With the vigorous development of new digital information technologies represented by the internet, the potential of the internet in promoting the transformation of agriculture towards green practices is becoming increasingly prominent. Research has shown that internet information has the ability to reduce and eliminate temporal and spatial barriers in information transmission [25], reduce search costs[26], as well as the function of sharing and disseminating new knowledge and technologies, and transforming traditional production structures. First, the internet integrates information, information elements, and material elements into a whole, enabling real-time integration of information senders, receivers, information, and information carriers [27]. This breaks the temporal and spatial limitations of information transmission, reduces information asymmetry, promotes efficient flow of advanced agricultural technology information to farmers, and optimizes resource allocation. Second, the internet promotes and applies advanced scientific and technological advancements in agricultural production, enhancing the technological proficiency and innovation capabilities of agricultural producers and enabling them to leverage advanced management concepts to generate higher economic benefits [28]. Third, the internet can reduce the costs of searching, acquiring, and negotiating, enabling access to comprehensive agricultural technologies and information. This, in turn, optimizes agricultural resource allocation and production structures, facilitating the transformation and upgrading of agricultural growth and improving production efficiency [29].

However, existing research still has the following deficiencies. First, in terms of research methods, the use of multinomial unordered logit regression cannot handle the endogeneity issue caused by self-selection in internet usage. The use of instrumental variable methods and propensity score matching approach, on the one hand, cannot address the issue of heterogeneity in treatment effects between internet users and non-internet users, and on the other hand, cannot handle the endogeneity issue caused by unobservable factors leading to omitted variable bias. Second, in terms of research content, although some scholars have studied the impact of the internet on farmers’ green production behavior, there are relatively few studies that explore the effects of the internet on farmers’ adoption of fertilizer reduction and efficiency enhancement technologies, as well as the underlying mechanisms involved. Therefore, based on the theoretical model of the internet’s influence on farmers’ adoption of fertilizer reduction and efficiency enhancement technologies, this study establishes a "counterfactual" analysis framework. It utilizes an endogenous switching probit model to examine whether internet usage can promote farmers’ adoption of fertilizer reduction and efficiency enhancement technologies. Furthermore, an intermediary effect model is employed to analyze the underlying mechanisms of how internet usage affects farmers’ adoption of fertilizer reduction and efficiency enhancement technologies.

The structure of this article is as follows. The first part is an introduction; The second part is theoretical analysis; The third part is data and variable analysis; The fourth part is model construction; The fifth part is empirical testing and analysis of impact mechanisms; The sixth part is the research conclusion and discussion.

### Theoretical analysis and research hypotheses

Farmers’ fertilizer application behavior is influenced by both internal and external factors, with the internet serving as an important external factor. As a platform for information dissemination and communication, the internet can provide farmers with relevant information and technical support on fertilizer reduction and efficiency enhancement techniques, thereby promoting their adoption of these techniques [30]. Firstly, the internet can provide farmers with abundant resources of fertilizer reduction and efficiency enhancement techniques, reducing the costs of information search, acquisition, and negotiation [31]. It breaks down information barriers, improves information dissemination efficiency, enhances farmers’ willingness to access information, deepens their understanding of fertilizer reduction and efficiency enhancement technologies, and increases the likelihood of adoption [32]. Secondly, the internet can provide online technical support to farmers, assisting them in solving challenges encountered in agricultural production. It can educate farmers on the proper utilization of fertilizer reduction and efficiency enhancement technologies, thereby improving the success rate and effectiveness of these techniques [33]. Lastly, the internet can f communication and collaboration among farmers, enabling them to share their experiences with fertilizer reduction and efficiency enhancement technologies [34]. This collaboration environment promotes the dissemination and impact of these technologies within the farming community [35].Therefore, the following assumption are proposed

#### Assumption 1

The use of the internet positively influences farmer’s adoption of fertilizer reduction and efficiency enhancement technologies.

When scrutinizing the impact of the internet on farmer’s adoption of fertilizer reduction and efficiency enhancement technologies, it is imperative to incorporate the notion of the "rational economic individual" as a logic basis. Rational economic individual is conceptualized depicted as individual who approaches economic decision-making in a rational manner, with their defining characteristic being the pursuit of maximum benefits while comprehensively considering various factors [36]. Research indicates that farmers, embodying the characteristics of rational economic individuals, typically engage in a comprehensive evaluation of expected benefits and potential risks when making production decisions.

During the planting process, farmers contemplate the potential outcomes of various decisions and calculate expected values by factoring in the probability and significance of these outcomes, all in pursuit of making the optimal decision [37]. Throughout this decision-making process, farmers also take into account the impacts of these outcomes on their benefits. By using the internet, farmers can attain a more precise understanding of the potential outcomes and corresponding benefits of different decisions. This equips them with the knowledge to make more informed decisions, consequently enhancing agricultural productivity. Expected benefits refer to the advantages that farmers envisage gaining through the adoption of fertilizer reduction and efficiency enhancement technologies [38]. Human motivation is dual, seeking both the maximization of economics benefits and non-economic benefits. Therefore, this article divides expected benefits into two dimensions: expected economic benefits and expected ecological benefits. Expected economic benefit pertains to the increase in income that farmers expect to gain by adopting fertilizer reduction and efficiency enhancement technologies [39]. Expected ecological benefit involves the anticipated improvement in the ecological environment expected by implementing fertilizer reduction and efficiency enhancement technologies. Therefore, as individuals driven by the pursuit of maximum benefits, farmers will proactively embrace fertilizer reduction and efficiency enhancement technologies when it promises substantial benefits [40].

As rational economic individual, farmers’ decisions may be influenced by factors such as costs and benefits, risks and uncertainties, personal preferences and values, as well as social and cultural influences. When farmers perceive a higher risk in adopting fertilizer reduction and efficiency enhancement technologies, the likelihood of generating benefits becomes smaller. Conversely, when they perceive a lower technological risk, the possibility of obtaining benefits from the technology increases [41]. In other words, farmers’ perception of technological risks can hinder their adoption of fertilizer reduction and efficiency enhancement technologies. However, farmers’ perception of risks associated with fertilizer reduction and efficiency enhancement technologies is based on subjective judgments formed through their experiences and acquired knowledge. The internet, as an important source of information, can facilitate farmers in accessing relevant information and acquiring technical knowledge about fertilizer reduction and efficiency enhancement technologies. This can help reduce their uncertainty and perception of risks in adopting such technologies, thereby increasing the likelihood of farmers adopting fertilizer reduction and efficiency enhancement technologies. In conclusion, the adoption behavior of farmers is a decision made by considering the trade-off between benefits and risks during the process of information assimilation. This paper proposes the following assumptions.

**Assumption 2.** The use of the internet has a positive impact on farmers’ adoption behavior, mediated by their expected returns.

### Assumption 2a

The use of the internet has a positive impact on farmers’ adoption behavior, mediated by their expected economic returns.

### Assumption 2b

The use of the internet has a positive impact on farmers’ adoption behavior, mediated by their expected ecological returns.

#### Assumption 3

The use of the internet can improve farmers’ perception of risk and promote their adoption of fertilizer reduction and efficiency enhancement technologies.

When farmers seek to access agricultural information through the internet, they should also consider the role of their social relationships and networks within their rural communities. This is because rural areas are relatively closed entities [42], and when an abundance of complex internet information is introduced into these areas, farmers may face significant challenges in learning new knowledge and technologies due to limitations in their technical skills and learning capabilities. Farmers are more likely to form similar thought patterns and approaches to action through social connections and interactions, which presents challenges in bridging the urban-rural digital divide. Therefore, this paper explores whether social networks play a moderating role in the relationship between internet usage and farmer’s adoption of fertilizer reduction and efficiency improvement technologies.

The role of social networks in the internet remains inconclusive. Some scholars argue that social networks can enhance the perception of usefulness and security, further strengthening the adoption of online internet information [43]. This helps bridge the "behavioral gap" between the internet and farmers’ technology adoption behavior and ultimately promotes the adoption of fertilizer reduction and efficiency enhancement technologies. In other words, social networks have a positive moderating effect in the influence of the internet [33]. Some scholars believe that the widespread and extensive information dissemination of the internet surpasses the temporal and spatial limitations of traditional social networks. They argue that social networks have a negative moderating effect in the influence of the internet. They suggest that internet channels for information acquisition can serve as substitutes for social networks [44].Therefore, this paper proposes assumption 4.

#### Assumption 4

There is a moderating effect of social networks in the process of internet influence on farmers’ adoption of fertilizer reduction and efficiency enhancement technologies.

**Fig 1:**
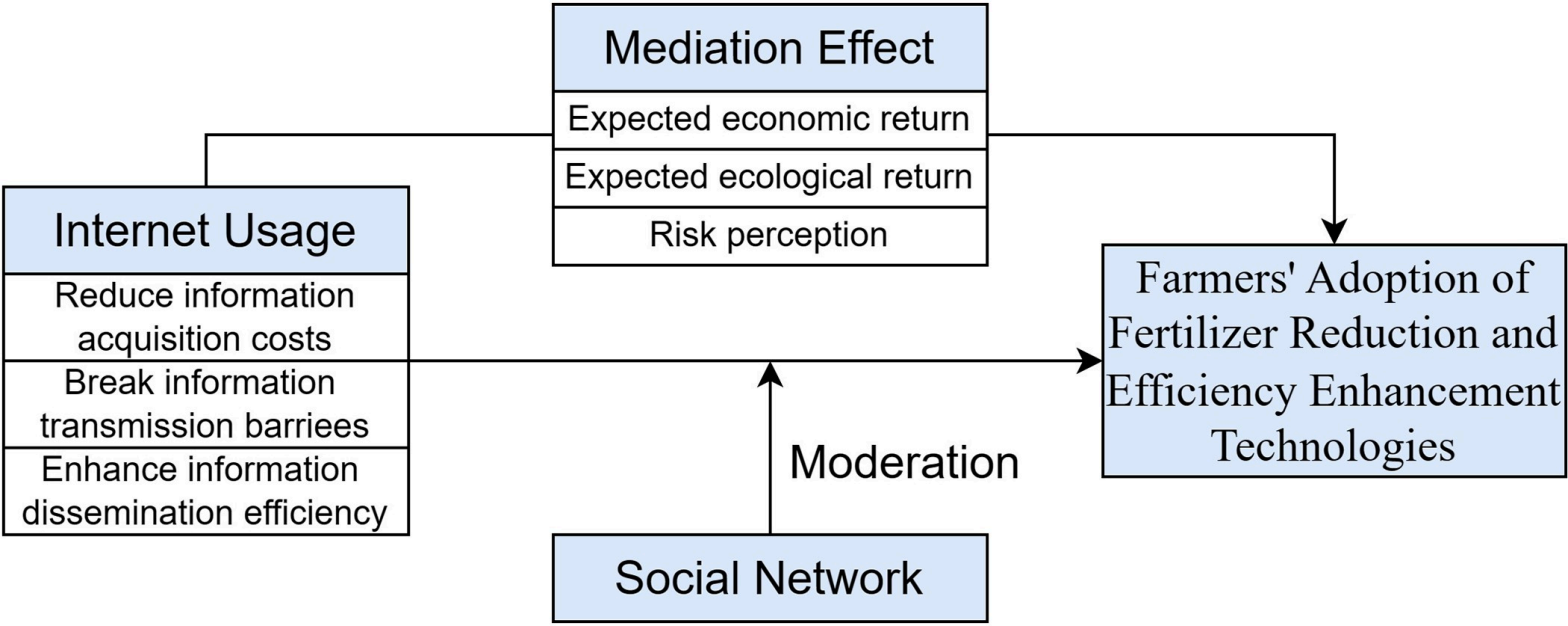
Mechanism of Internet Usage on Farmers’ Adoption of Fertilizer Reduction and Efficiency enhancement Technologies.

In conclusion, the internet has a significant influence on farmers’ adoption of fertilizer reduction and efficiency enhancement technologies. Based on this, the theoretical framework constructed in this article (refer to Fig 1) is as follows.

## Materials and methods

### Materials

#### Data source

The data used in this study were collected from a one-year household survey conducted by the research team in Henan Province starting from March 2022. The reason for selecting Henan Province as the survey target is. Firstly, Henan Province is a major agricultural province in China with a high usage of fertilizers. From 2017 to 2022, the annual fertilizer usage in Henan Province has consistently remained above 6 million tons, ranking first in the country. The excessive use of fertilizers is a significant issue in the province. Secondly, agriculture in Henan Province exhibits a diverse range of production methods. Due to the traditional contradiction of having a large population in a limited land area, there still exist small-scale farming operations. However, with the implementation of land leveling measures and the shortage of labor due to population outflow, there has also been a trend towards large-scale mechanized production in agricultural activities. Additionally, driven by urbanization and the presence of low-lying hilly areas, there has also been an emergence of non-grain agricultural management practices. Therefore, as a research area, the diversity of production methods in Henan Province can provide us with multiple perspectives and multilevel analysis. The investigation adopts a combination of stratified step-by-step sampling and random sampling methods.

Henan Province, as a major agricultural province in China, covers a geographical area of 167,000 square kilometers and has a population of as high as 98.72 million. The province’s arable land area exceeds 110 million mu, ranking third nationwide. Given the large population and abundant land resources in Henan Province, this survey adopts a sampling method for effective implementation. Although conducting a large-scale survey presents certain challenges, it is also one of our future directions for improvement. Moreover, Henan Province has diverse topography, and the main crops are planted in plain and hilly areas. In order to reduce regional bias, we deliberately selected two plain areas and two hilly areas as research samples. This strategy helps to more accurately grasp the differences between different regions, so as to more comprehensively understand the situation and behavior of farmers. The sampling process in this study pays close attention to factors such as terrain differences and farming practices, aiming to minimize regional bias or variations among agricultural areas as much as possible. Specifically, we selected four cities in Henan Province: Luoyang, Zhoukou, Xuchang, and Xinyang. These cities exhibit diversity and uniqueness in terms of terrain and farming practices. In terms of terrain, Zhoukou and Xuchang are predominantly plains, while Luoyang and Xinyang are characterized by mountains and hills. Regarding farming practices, Zhoukou and Xuchang primarily engage in meticulous farming, focusing on wheat and corn cultivation. Luoyang, due to its rugged terrain, mainly focuses on cash crops. Meanwhile, Xinyang, located in the south, specializes in rice cultivation. The differences in terrain and the diversity and distinctiveness of production methods indicate that the four selected cities are representative choices. Each city selects 1–2 counties (districts), and each county (district) randomly selects 4–5 townships. Each township randomly selects 4–6 villages, and each village randomly selects 40 farm households. Based on the research topic and relevant indicators, invalid questionnaires are removed, resulting in a total of 1295 valid questionnaires.

#### Variable description

Dependent variable. Adoption behavior of fertilizer reduction and efficiency enhancement techniques. Based on the actual rural situation, the following question is designed. "In agricultural production activities, do you adopt fertilizer reduction and efficiency enhancement techniques, such as using green manure or organic fertilizer, and other environmentally friendly techniques?" The question is set as a 2-point scale. Assign a value of 1 for "yes" and a value of 0 for "no".

Core independent variable. The focus of this article is whether the use of the Internet by farmers affects their adoption of fertilizer reduction and efficiency enhancement techniques. Therefore, Internet usage is the core independent variable. The following question is designed. "How do you typically obtain agricultural information?" When a farmer chooses to use mobile phones and online information, it is considered that the farmer uses the Internet to access information, and a value of 1 is assigned. Otherwise, a value of 0 is assigned. Descriptive analysis of farmers’ adoption behavior of fertilizer reduction and efficiency enhancement techniques are presented in Table 1.

**Table 1:** Descriptive analysis of farmers’ adoption behavior of fertilizer reduction and efficiency enhancement techniques.

Internet usage	Observation	Percentage (%)	Adopting technology		Non-adopting technology	
			Observation	Percentage (%)	Observation	Percentage (%)
Internet-using farmers	688	53.13	438	63.66	250	36.34
Non-Internet-using farmers	607	46.87	341	56.18	266	43.82
Total	1295		779	60.15	516	39.85

Other explanatory variables. Building on the findings of Jiang Weijun [45] and Gao Tianzhi [46], this study selects various variables as control variables, including the respondents’ gender, age, education level, party membership, employment status, family annual income, total family population, land area, distance from the village to the city, environmental awareness, level of internet infrastructure development, level of trust in friends and neighbors, and level of trust in the government.

Mediating variables. Expected economic benefits refer to the farmers’ anticipation of economic income after implementing fertilizer reduction and efficiency enhancement techniques. Therefore, based on the rural context, the item "Do you believe that adopting fertilizer reduction and efficiency enhancement techniques in green agriculture can bring in more economic income?" is used to reflect this indicator. The item is designed using a Likert five-point scale, with the farmers’ perceptions categorized as "strongly disagree, disagree, neutral, agree, strongly agree," assigned values ranging from 1 to 5. Expected ecological benefits primarily refer to the improvement of agricultural ecological environment and ecosystems through the implementation of fertilizer reduction and efficiency enhancement techniques. Therefore, based on the rural context, the item "Do you believe that adopting fertilizer reduction and efficiency enhancement techniques in green agriculture can alleviate ecological environmental pressure?" is used to reflect this indicator. The item is designed in the same manner as described above, using a Likert five-point scale. Risk perception refers to the subjective judgment of farmers regarding the risks associated with fertilizer reduction and efficiency enhancement techniques. Therefore, based on the rural context, the item "Do you believe that you can bear the risks associated with adopting green production methods?" is used to reflect this indicator. The item is designed using a Likert five-point scale, where farmers’ perceptions are categorized into five levels. "strongly disagree, disagree, neutral, agree, strongly agree," assigned values ranging from 1 to 5.

Moderating variables. Taking the variable "whether there are friends in the farmer’s WeChat contacts who have adopted fertilizer reduction and efficiency enhancement techniques" as a social network variable, "yes" is assigned a value of 1, while "no" is assigned a value of 0."WeChat friends" includes both close friends and relatives in farmers’ offline "strong tie" social networks and online friends in the "weak tie" social networks, breaking through the limitations of previous studies that rely on traditional interpersonal networks to measure social networks. Since introducing interaction terms between social networks and internet usage in the econometric model may lead to multicollinearity issues, a centralization process was applied to internet usage and social network variables before conducting moderation effect analysis to weaken the collinearity between individual variables and variable interactions.

Exclusion constraint variable. In the application of endogenous switching models, it is necessary to include exclusion constraint variables, which are instrumental variables that affect the selection equation but not the outcome equation[47]. In this study, "the number of contacts in your mobile phone" is chosen as the instrumental variable. The primary rationale behind this selection is that when farmers have a larger number of contacts in their mobile phones, it indicates frequent usage of mobile phones for communication or information retrieval purposes. Farmers are more inclined to utilize mobile phones as a channel for accessing network information. Importantly, this variable does not directly influence farmers’ proactive adoption behavior. In this paper, we conducted an assessment of the validity of instrumental variables, and the results of this validation are detailed in the manuscript. Treating internet usage as the dependent variable, with instrumental variables and other control variables as independent variables, we employed Endogenous Switching probit models. The coefficient associated with the variable " the number of contacts in your mobile phone " was found to be 0.103, with a corresponding p-value of 0.000. This suggests that the " the number of contacts in your mobile phone " significantly influences farmers’ use of the internet to obtain information. In other words, as the number of contacts in a farmer’s mobile phone increases, the probability of the farmer using the internet also increases. This result passed the significance test, meeting the requirements for instrumental variable relevance. In assessing the validity of instrumental variables, we treated farmers’ adoption behavior as the dependent variable, with internet usage, instrumental variables, and other control variables as independent variables. Using probit regression models, we found that the coefficient associated with the variable "number of contacts in mobile phone" was 1.37, with a corresponding p-value of 0.31. This indicates that the variable " the number of contacts in your mobile phone " does not directly influence farmers’ adoption behavior of fertilizer reduction and efficiency enhancement technologies. Therefore, this result confirms the exogeneity condition of the instrumental variable. Therefore, based on the empirical test results, the variable " the number of contacts in your mobile phone " largely meets the requirements of instrumental variable relevance and exogeneity. Thus, it can be considered that the instrumental variable chosen in this study is effective. The definitions and descriptive statistics of the variables are presented in Table 2.

**Table 2:** Variable definition and descriptive statistics.

Variables	Definition	Non-internet usage		Internet usage	
		Mean	S.D.	Mean	S.D.
Internet usage	How do you typically obtain agricultural information? Using a mobile phone and internet to access information = 1, Using other means to obtain information = 0.				
Adopt fertilizer reduction and efficiency enhancement technology	In agricultural production activities, do you employ environmentally friendly techniques such as adopt fertilizer reduction and efficiency enhancement technology and using green manure or farm manure? Yes = 1, No = 0.	0.562	0.497	0.637	0.481
Number of mobile phone contacts	What is the number of contacts in your mobile phone? Less than 50 = 1, 50–100 = 2, 100–150 = 3, 150–200 = 4, more than 200 = 5.	2.000	1.177	2.196	1.256
Age	Under 30 years old = 1,30–40 years old = 2, 41–50 years old = 3,51–60 years old = 4,60 years old = 5	2.753	1.488	1.866	1.129
Gender	Male = 1, female = 0	0.486	0.500	0.408	0.492
Educational level	Primary school and below = 1, middle school = 2, high school and technical secondary school = 3, junior college = 4, Bachelor’s degree and above = 5	2.712	1.545	3.660	1.418
Party member	Yes = 1, no = 0	0.206	0.405	0.298	0.458
Part-time employment	Non-farm employment = 1, full-time farming = 2, part-time farmers = 3	2.069	0.818	1.888	0.938
Annual household income	Less than 10,000 RMB = 1, 10,000–30,000 RMB = 2, 40,000–60,000 RMB = 3, 70,000–100,000 RMB = 4, more than 100,000 RMB = 5.	2.468	1.175	2.577	1.143
Total population of the family	The actual number of rural household population	4.936	1.693	4.793	1.490
Acres of land	0-3acres of land = 1,4-6acres of land = 2,7-10acres of land = 3,11-15acres of land = 4,over 15acres of land = 5	2.076	1.093	1.882	1.109
The distance between the village and the city	How far is your village from the county? 3 km and below = 1,4–6 km = 2, 7–10 km = 3,11–20 km = 4, more than 20 km = 5	3.000	1.386	2.754	1.425
Environmental awareness	How much do you agree with being concerned about environmental issues encountered during agricultural production? Strongly disagree = 1, Somewhat disagree = 2, Neutral = 3, Somewhat agree = 4, Strongly agree = 5.	3.367	0.826	3.353	0.823
Network facility	How would you rate the degree of infrastructure development in your location’s network? Very inadequate = 1, Somewhat inadequate = 2, Neutral = 3, Somewhat adequate = 4, Very adequate = 5.	3.293	0.850	3.368	0.832
Confidence	How would you rate your level of trust in your relatives, friends, and neighbors? Very distrustful = 1, Somewhat distrustful = 2, Neutral = 3, Somewhat trusting = 4, Very trusting = 5.	3.600	0.777	3.573	0.773
	How would you rate your level of trust in policies and regulations? Very distrustful = 1, Somewhat distrustful = 2, Neutral = 3, Somewhat trusting = 4, Very trusting = 5.	3.712	0.838	3.746	0.84
Risk perception	How willing are you to accept the risks associated with adopting fertilizer reduction and efficiency-increasing technologies? Strongly disagree = 1, Somewhat disagree = 2, Neutral = 3, Somewhat agree = 4, Strongly agree = 5.	2.840	0.852	3.025	0.784
Expected returns	Do you believe that implementing fertilizer reduction and efficiency-increasing technologies in green agricultural cultivation can generate more economic income? Strongly disagree = 1, Somewhat disagree = 2, Neutral = 3, Somewhat agree = 4, Strongly agree = 5.	3.389	0.796	3.538	0.823
	Do you believe that implementing fertilizer reduction and efficiency-increasing technologies in green agricultural cultivation can alleviate ecological environmental pressures? Strongly disagree = 1, Somewhat disagree = 2, Neutral = 3, Somewhat agree = 4, Strongly agree = 5.	3.517	0.826	3.644	0.83
Social network	Do any of your friends adopt fertilizer reduction and efficiency-increasing technologies? Yes = 1, no = 0	0.226	0.418	0.414	0.493

### Model description

#### Endogenous switching probit model

As limited rational individuals, farmers’ decision-making behavior is to some extent the pursuit of the optimal "self-selection" result[48–50]. Whether farmers choose to use the internet is influenced by many factors, which may also simultaneously affect farmers’ adoption of reduced fertilizer and increased efficiency technologies[51]. Therefore, when estimating the impact of Internet use on the adoption behavior of farmers’ fertilizer reduction and efficiency increase technology, this paper selected Endogenous Switching probit model to fit the dependence relationship between the conversion equation (whether the Internet is used) and the result equation (whether the fertilizer reduction and efficiency increase technology is adopted), so as to weaken the endogeneity problem [52,53].

Furthermore, the Endogenous Switching probit model can be used to address endogeneity issues caused by omitted variables or reverse causality, as well as control for bias caused by unobservable factors [54,55]. Moreover, the Endogenous Switching model not only effectively resolves sample selection bias caused by unobservable factors, but also fits equations for the adoption of fertilizer reduction and efficiency enhancement technologies by farmers who use the internet and those who do not, as well as counterfactual analysis, to some extent compensating for the limitations of methods such as propensity score matching [56,57]. Therefore, this study selects the Endogenous Switching model to address endogeneity issues in the baseline regression.

Finally, the direct evaluation of the in pact of internet usage on farmers’ adoption of fertilizer reduction and efficiency enhancement technologies is not possible because it is not possible to observe the same farmer simultaneously in both internet usage and non-usage states. In order to address the aforementioned issues, this study employs an endogenous switching probit model and constructs a counterfactual analysis framework based on the regression results to estimate the treatment effect of internet usage on the probability of farmers adopting fertilizer reduction and efficiency enhancement technologies.

First, the use of the internet by farmers is taken as the treatment variable (T_i_). If the internet is used, T_i_ = 1; otherwise, T_i_ = 0. The internet usage by farmers can be represented as.

$$T_{i}T_{i}=Z_{i}^{\prime }\;\gamma +\mu_{i},T_{i}=\left\{ \begin{array}{c} 1,T_{i}>0\\ 0,T_{i}⩽0 \end{array}\right.$$ (1)

Next, the outcome equation for farmers’ adoption of fertilizer reduction and efficiency enhancement technologies under different states is defined as.

$$y_{i}=\{\begin{array}{c} y_{1i},T_{i}=1,y_{1i}=1(y_{1i}^{*}>0),y_{it}^{*}=X_{it}^{\prime }\;\beta_{t}+\sigma_{\mu t}\lambda_{it}+\varepsilon_{it}\\ y_{0i},T_{i}=0,y_{0i}=0(y_{0i}^{*}>0),y_{iu}^{*}=X_{iu}^{\prime \prime }\;\beta_{u}+\sigma_{\mu u}\lambda_{iu}+\varepsilon_{iu} \end{array}$$ (2)

y_1i_ and y_0i_ represent the latent variables of farmers’ adoption of fertilizer reduction and efficiency enhancement technologies when using or not using the internet, respectively. They determine the observed binary state variables y_1i_ and y_0i_, which represent the adoption behavior of fertilizer reduction and efficiency enhancement technologies. $X_{it}^{\prime }$ and $X_{iu}^{\prime \prime }$ are covariates that influence farmers’ adoption of fertilizer reduction and efficiency enhancement technologies. β_t_ and β_u_ are the coefficients to be estimated.

Estimating the probability of farmers using the internet and adopting fertilizer reduction and efficiency enhancement technologies using the normal distribution function.

$$E(Y_{it}|T_{i}=1,X=x)=X_{it}^{\prime }\;\beta_{t}+\sigma_{\mu t}\lambda_{it}$$ (3)

The probability of farmers using the internet and not adopting fertilizer reduction and efficiency enhancement technologies is.

$$E(Y_{it}|T_{i}=1,X=x)=X_{it}^{\prime }\;\beta_{u}+\sigma_{\mu u}\lambda_{it}$$ (4)

The probability of farmers not using the internet and adopting fertilizer reduction and efficiency enhancement technologies is.

$$E(Y_{iu}|T_{i}=0,X=x)=X_{iu}^{\prime }\;\beta_{u}+\sigma_{\mu t}\lambda_{iu}$$ (5)

The probability of farmers not using the internet and not adopting fertilizer reduction and efficiency enhancement technologies is.

$$E(Y_{iu}|T_{i}=0,X=x)=X_{iu}^{\prime }\;\beta_{t}+\sigma_{\mu t}\lambda_{iu}$$ (6)

The average treatment effect (ATT_i_) of internet usage on farmers’ adoption behavior can be expressed as the difference between Eqs (3) and (4).

$$\begin{array}{c} ATT_{i}=E(Y_{it}|T_{i}=1,X=x)-E(Y_{iu}|T_{i}=1,X=x)\\ =X_{it}^{\prime }\left(\beta_{t}-\beta_{u}\right)+\lambda_{it}\left(\sigma_{\mu t}-\sigma_{\mu u}\right) \end{array}$$ (7)

The average treatment effect (ATU_i_) of non-internet usage on farmers’ adoption behavior can be expressed as the difference between Eqs (5) and (6).

$$\begin{array}{c} ATU_{i}=E(Y_{it}|T_{i}=0,X=x)-E(Y_{iu}|T_{i}=0,X=x)\\ =X_{iu}^{\prime }\left(\beta_{t}-\beta_{u}\right)+\lambda_{iu}\left(\sigma_{\mu t}-\sigma_{\mu u}\right) \end{array}$$ (8)

In conclusion, this study examines the average treatment effect of internet usage on farmers’ adoption behavior of fertilizer reduction and efficiency enhancement technologies using the mean values of ATT_i_ and ATU_i_.

### Mediation model

The mediation effect of expected benefits and risk perception is examined in this study using the sequential testing method. The formulation of the mediation effect model is as follows.

$$Y=cX+\varepsilon_{1}$$ (9)

$$M=\alpha X+\varepsilon_{2}$$ (10)

$$Y=\beta^{\prime }X+\gamma M+\varepsilon_{3}$$ (11)

In the mediation effect model of this study, the mediator variable M represents expected economic benefits, expected ecological benefits, and risk perception. The dependent variable Y represents farmers’ adoption behavior, and X represents internet usage. As Y is a binary variable, the coefficients in Eqs (9) and (11) represent the marginal utility at the mean values.

### Moderation model

Construct the following model to examine the moderation effect of social networks on the influence of the internet in the adoption of green agricultural technologies by farmers.

$$Y=\beta_{1}C_{i}+\beta_{2}V_{i}+\beta_{3}C_{i}V_{i}+\beta_{4}Z_{i}+\lambda_{i}$$ (12)

In the equation, Y represents farmers’ adoption behavior of fertilizer reduction and efficiency enhancement technologies, C refers to internet usage, V represents the moderating variable of social networks, and the interaction term CV represents the moderating effect of social networks on the relationship between internet usage and the adoption of fertilizer reduction and efficiency enhancement technologies. Z represents the control variables, and β_k_(k = 1~4) denotes the coefficients to be estimated, while, λ_i_ represents the error term.

### Result analysis

#### Endogenous switching probit model result

In this study, the core variable is whether farmers use the internet, and the outcome variable is the adoption behavior of fertilizer reduction and efficiency enhancement technologies. The model (1) and (2) were estimated using the full information maximum likelihood method, and the regression results are shown in Table 3.

**Table 3:** The combined estimation results of using an internet decision model and farmer adoption behavior model.

Variables	Internet usage	Adoption behavior	
		Use the Internet	Not using the Internet
Number of mobile phone contacts	0.1034*** (0.2947)		
Age	-0.2815*** (0.5804)	0.2117*** (0.7744)	0.0265 (0.8044)
Gender	-0.5072 (0.0850)	0.0701 (0.1033)	-0.991 (0.1001)
Education level.	0.1029** (0.5141)	0.0034 (0.0661)	0.1853*** (0.0660)
Party member	0.1617* (0.0948)	-0.4554 (0.1051)	-0.0595 (0.1340)
Part-time employment	0.0702 (0.0501)	0.1293** (0.0595)	0.2362*** (0.0664)
Annual household income	0.0959** (0.0379)	-0.1299*** (0.0461)	-0.0485 (0.0557)
Total household population	-0.0203 (0.0307)	-0.0287 (0.0362)	0.0176 (0.0374)
Acres of land	0.1027** (0.0411)	0.0744 (0.0487)	-0.0225 (0.0517)
The distance between the village and the city	0.0225 (0.0315)	-0.1155*** (0.3851)	-0.7578* (0.0415)
Environmental awareness	-0.0065 (0.0546)	0.0696** (0.0656)	0.0094* (0.0649)
Network facility	0.0970* (0.0601)	-0.1372 (0.7844)	0.0070 (0.0701)
Level of trust in friends and neighbors.	-0.6413 (0.0636)	0.0470 (0.0817)	-0.0132 (0.0726)
Level of trust in government	0.0924 (0.0582)	0.1982*** (0.0745)	0.2423*** (0.0702)
Constant	-0.5612 (0.4263)	0.5643 (0.5363)	
rho1		0.9997 (0.0349)	
rho0			-0.8765 (0.1675)
Wald chi2(17) = 172.71*** LR test of indep. eqns.(rho1 = rho0 = 0). chi2(2) = 5.09 prob > chi2 = 0.0785 Observations 1295 688 607			

Note

*, ** and *** indicate significance at the 10%, 5% and 1% levels, respectively.

As shown in Table 3, the Wald chi-square value is significant at the 1% level, indicating that the overall model is statistically significant. Additionally, the significance of rho1 at the 5% level suggests the presence of unobservable factors that simultaneously affect farmers’ internet usage and their adoption of fertilizer efficiency technologies. This implies that the baseline regression model may suffer from selection bias, and using an endogenous switching model is appropriate. The estimated value of rho1 being positive indicates that farmers who use the internet have a higher probability of adopting fertilizer efficiency technologies compared to the average level of farmers in the sample. On the other hand, the value of rho0 being negative suggests that farmers who do not use the internet have a lower probability of adopting fertilizer efficiency technologies compared to the average level of farmers in the sample.

Factors such as age, level of education, party membership, household annual income, land area, level of internet infrastructure, and number of mobile contacts significantly influence farmers’ internet usage. Specifically, age is found to be significant at the 1% level, and it has a negative effect. This suggests that younger individuals are more inclined to use the internet for social activities and obtaining external information. It indicates a shift in social patterns from "relational-based socialization" to "stranger-based socialization. "The level of education is found to be significant at the 5% level, and it has a positive effect. This indicates that, on one hand, farmers with higher education levels are more aware of the value of internet information for agricultural production and are more willing to understand advanced production technologies and market information. On the other hand, the improvement in education level helps farmers bridge the "digital divide" and become proficient in using the internet. Party membership is found to be significant at the 10% level, and it has a positive effect. This suggests that as rural "talents," party members generally have higher social and economic status as well as broader perspectives. They are more likely to engage in frequent exchanges of information with the outside world. Additionally, the vigorous movement of digital rural development has also facilitated deeper access to internet information for grassroots party organizations in rural areas. Household annual income is found to be significant at the 5% level, and it has a positive effect. This suggests that there may be a feedback mechanism between household income and internet usage. On one hand, using the internet helps farmers acquire market information and knowledge of production techniques, thus improving agricultural productivity and diversifying income sources. This, in turn, contributes to an increase in the household income level. On the other hand, an increase in household income means that individuals have the means to afford internet devices, thereby enhancing their access to internet information. Land area per mu is found to be significant at the 5% level, and it has a positive effect. This is likely because larger-scale farmers, who often achieve scale operation through land transfer, are more inclined to use the internet to access advanced crop management practices, agricultural production technologies, and market trading information. This is especially important given the high costs of land transfer and the instability of agricultural income. The level of internet infrastructure development is found to be significant at the 10% level, and it has a positive effect. This indicates that the rapid development of agricultural internet infrastructure and the widespread application of digital technologies in agricultural production have contributed to the increased internet participation of farmers and helped bridge the digital divide. The number of mobile phone contacts is found to be significant at the 1% level. This may be because the quantity of mobile phone contacts is closely related to the complexity of farmers’ social networks. The larger the number of mobile phone contacts, it signifies that farmers’ social networks may extend beyond the "acquaintance-based" society in rural areas and reach urban regions. This close interaction between rural and urban areas encourages farmers to access information through the internet.

Significant disparities exist in the factors influencing the adoption of fertilizer reduction and efficiency enhancement technologies between farmers who use the internet and those who do not. In terms of individual characteristics, age only has a significant positive effect on farmers who use the internet, while its influence on farmers who do not use the internet is not statistically significant. This suggests that older farmers’ social networks rely more on social interactions within the village, and internet information can effectively compensate for their limited sources of information. By utilizing internet information to optimize their production techniques and fertilizer application choices, it can enhance their motivation to adopt fertilizer reduction and efficiency-increasing technologies. Education level only has a significant positive effect on farmers who do not use the internet, while its influence on farmers who use the internet is not statistically significant. This may be because, on the one hand, farmers with higher education levels, even if they do not use the internet, have developed environmental awareness through their educational process, which helps them recognize the environmental value of adopting fertilizer reduction and efficiency-increasing technologies, making them more willing to adopt such technologies. On the other hand, for farmers with lower education levels, internet information can effectively enhance environmental awareness and technological knowledge through case sharing and technology dissemination. This, in turn, helps farmers adopt fertilizer reduction and efficiency-increasing technologies.

In terms of production characteristics, having concurrent business has a significant positive impact on farmers. This indicates that regardless of whether they acquire information through the internet or not, having concurrent business contributes to farmers’ adoption of fertilizer reduction and efficiency-increasing technologies. This may be because, on the one hand, farmers gain information through their concurrent business and achieve diversified household income, which increases the likelihood and feasibility of adopting fertilizer reduction and efficiency-increasing technologies. On the other hand, having concurrent business replaces the farming labor time with non-farming labor time, reducing the labor time spent on field management. This leads to a reduction in agricultural inputs such as fertilizer and pesticides, thereby promoting farmers to adopt fertilizer reduction and efficiency-increasing technologies. Household annual income only has a significant negative effect on internet users, while its influence on non-internet using farmers is not statistically significant. This indicates that internet information provides new choices for farmers’ production decisions, especially for farmers with lower household annual income, who are more willing to adopt fertilizer reduction and efficiency-increasing technologies to reduce production costs.

In terms of location characteristics, the distance between villages and cities has a significant negative impact on farmers. This may be due to the fact that, on the one hand, villages located farther from cities generally have relatively poor socio-economic and digital conditions. The internet infrastructure may not be well-developed, and farmers in these areas may have less willingness to adopt new planting technologies. In contrast, farmers closer to cities are more likely to be exposed to emerging agricultural production technologies and environmental information. Hence, they are more willing to adopt fertilizer reduction and efficiency-increasing technologies. On the other hand, the demand for green and organic agricultural products from urban residents drives farmers in the vicinity of cities to adopt fertilizer reduction and efficiency-increasing technologies. This is driven by their economic interests in reducing chemical pollution in the production process of agricultural products.

In terms of cognitive characteristics, environmental awareness only has a significant negative effect on internet users, while its influence on non-internet using farmers is not statistically significant. This indicates that internet information can effectively bridge the gap between farmers’ willingness and behavior in adopting fertilizer reduction and efficiency-increasing technologies. It can guide farmers in effectively practicing these technologies. However, it may not effectively cultivate farmers’ environmental awareness and enhance their willingness to proactively adopt fertilizer reduction and efficiency-increasing technologies. The level of trust in the government has a significant positive impact on farmers. This may be because the government plays an irreplaceable role in the process of agricultural technology promotion. The level of trust in the government determines whether farmers are willing to learn, understand, and adopt new technologies, which indirectly affects their willingness to adopt fertilizer reduction and efficiency enhancement techniques.

#### The average treatment effect

Calculating the average treatment effect of using the internet on farmers’ adoption behavior of fertilizer reduction and efficiency enhancement techniques using Eqs (7) and (8) (refer to Table 4). In Table 4, (a) and (b) correspond to the adoption behavior of sample farmers when using and not using the internet, respectively, which are represented by Eqs (3) and (5). (c) and (d) correspond to Eqs (4) and (6), respectively. Specifically, (c) and (d) represent counterfactual outcomes.

**Table 4:** Average treatment effect of internet usage on farmers’ adoption behavior.

Category of farmers	Use the Internet	Not using the Internet	ATT	ATU
Internet-using farmers	(a)0.9841	(c)0.7077	0.2764***	
Non-internet-using farmers	(d)0.9750	(b)0.6931		0.2819***

Overall, the use of the internet has a positive and significant impact on farmers’ adoption of fertilizer reduction and efficiency enhancement practices, at a significance level of 1%. The estimated results of ATT indicate that for farmers who have already used the internet, not using the internet would lead to a decrease of 28.09% in the probability of adopting fertilizer reduction and efficiency enhancement practices. The estimated results of ATU indicate that if farmers who do not use the internet to access information are able to use the internet for information acquisition, the probability of adopting fertilizer reduction and efficiency enhancement practices would increase by 40.67%. This further demonstrates that the use of the internet can effectively promote farmers’ adoption of fertilizer reduction and efficiency enhancement technologies.

#### The mediation effect

The estimated results from regression (1) in Table 5 demonstrate that internet usage has a significant promoting effect on farmers’ adoption behavior, further confirming the previous analysis. The regression results (2) indicate that internet usage significantly increases farmers ’ expected ecological benefits. Regression (3) shows that an increase in expected ecological benefits significantly promotes farmers’ adoption of fertilizer reduction and efficiency enhancement technologies, which aligns with the Assumption 2b of this study. By comparing regression (1) and regression (3), it is found that after adding the mediator variable, the coefficient of internet usage on farmers’ adoption of fertilizer reduction and efficiency enhancement technologies decreases from 0.1036 to 0.0994. This indicates that the effect of internet usage on farmers’ adoption behavior is partially mediated by expected ecological benefits. Therefore, the aforementioned influence mechanism is validated.

**Table 5:** Analysis of the impact mechanisms of internet usage on farmer adoption behavior.

Project	Expected ecological returns			Expected economic returns			Risk perception		
	regression(1) Adoption behavior	regression(2) Expected ecological returns	regression(3) Adoption behavior	regression(4) Adoption behavior	regression(5) Expected economic returns	regression(6) Adoption behavior	regression(7) Adoption behavior	regression(8) Risk perception	regression(9) Adoption behavior
Internet usage	0.1036*** (0.0290)	0.1267*** (0.0438)	0.0994*** (0.0291)	0.1036*** (0.0290)	0.1383*** (0.0441)	0.0993*** (0.0291)	0.1036*** (0.0290)	0.1086*** (0.0459)	0.0995*** (0.0290)
Expected ecological returns			0.0328*(0.0185)						
Expected economic returns						0.0321* (0.0184)			
Risk perception									0.0372** (0.0177)
Control variables	Controlled	Controlled	Controlled	Controlled	Controlled	Controlled	Controlled	Controlled	Controlled

From the regression results (5) and (6), it is evident that internet usage significantly enhances farmers’ expectations of economic returns. This indicates that farmers can access a wealth of information and knowledge through the internet, leading to a heightened awareness of the economic benefits associated with adopting fertilizer reduction and efficiency enhancement technologies. Anticipated economic returns play a crucial role in positively driving farmers’ adoption behaviors. This further corroborates the positive impact of the internet in the agricultural sector. Economic gain serves as a potent incentive for farmers, and it is evident that a portion of the influence of internet usage on farmers’ adoption behavior is realized through their expectations of economic returns. Thus, the aforementioned mechanism is substantiated.

From the regression results (8) and (9), it is evident that internet usage can significantly reduce farmers’ perception of the risks associated with fertilizer reduction and efficiency enhancement technologies. This indicates that, with the assistance of the internet, farmers can gain a clearer understanding of the advantages and potential benefits of these technologies. Through channels such as social media or news, they can access shared experiences from agricultural experts or other farmers, learning from successful case studies. These factors help alleviate farmers’ perception of the risks associated with adopting new technologies, ultimately encouraging them to experiment with these innovations and make changes. Therefore, internet usage can influence farmers’ adoption of fertilizer reduction and efficiency enhancement technologies through the pathway of risk perception, and this mechanism has been validated.

#### The moderation effect

According to the regression results in Table 6, it can be concluded that social networks have a significant positive impact on farmers’ adoption of fertilizer reduction and efficiency technologies, and this result has passed the significance test at the 1% level. This indicates that farmers are more likely to adopt fertilizer reduction and efficiency enhancement technologies when they have friends in their WeChat contact list who have already adopted such technologies. The coefficient of the interaction term between internet usage and social network is -0.2366, which is significant at the 10% level. This indicates that social networks can negatively moderate the impact of internet usage on farmers’ adoption behavior of fertilizer reduction and efficiency enhancement technologies. Internet usage helps farmers with weaker social network ties to adopt new technologies. However, as farmers’ social network ties gradually strengthen, the impact of internet usage on their adoption of fertilizer reduction and efficiency enhancement technologies. Both internet usage and social networks have a significant positive impact on farmers’ adoption behavior of fertilizer reduction and efficiency technologies. Furthermore, the coefficient of internet usage is significantly larger than that of social networks, indicating that internet usage can serve as a substitute for social networks in influencing farmers’ adoption of fertilizer reduction and efficiency technologies.

**Table 6:** Test of the moderating effects of social networks.

	Chemical fertilizer reduction and efficiency enhancement technologies	
Variables	Coefficient	Standard deviation
Internet usage	0.1085***	0.0345
Social Networks	0.0313*	0.0481
Internet usage ×social network	-0.2366*	0.0605
Control variables	Controlled	Controlled

### Robustness check

#### Robustness test 1: Alternative estimation method

To further validate the accuracy of the results, a robustness test was conducted using propensity score matching. Firstly, the probability of farmers adopting fertilizer reduction and efficiency enhancement technologies through internet usage was calculated based on the formula for ATT (Average Treatment Effect on the Treated). Table 7 displays the calculated probabilities using three different matching methods. It can be observed that the results from the three matching methods are quite similar and all significant at the 1% level. This suggests that the ATT values exhibit a strong robustness.

**Table 7:** Results of effects estimation.

Matching method	Experimental group	Control group	Average treatment effect	T
Nearest Neighbour Matching	0.6366	0.4797	0.0791*** (0.0280)	3.95
Caliper matching	0.6366	0.5023	0.1090*** (0.0325)	4.11
Nuclear matching	0.6366	0.5264	0.1044*** (0.0309)	3.58
Average	0.6366	0.5028	0.0975	

The average value of ATT calculated using different matching methods indicates that farmers who use the internet have a higher probability of adopting the technology. In other words, under the condition of excluding other influencing factors, farmers using the internet have a 9.75% higher adoption efficiency compared to those who do not use the internet. This aligns with the results obtained from the endogenous switching probit model analysis. It is evident that internet usage has become a significant driving force in promoting the adoption of fertilizer reduction and efficiency enhancement technologies among farmers.

Furthermore, after calculating the probability of farmers adopting fertilizer reduction and efficiency enhancement technologies through internet usage, to further validate the empirical results of this paper, a balance test using propensity score matching needs to be conducted. This test examines whether the variables become balanced between the two groups after matching. As shown in Table 8, the Pseudo-R^2^ significantly decreased from 0.100 before matching to 0.003~0.009; the LR statistic dropped significantly from 179.17 to 6.15~16.33; the significance test p-values changed from highly significant to not significant; the mean bias decreased from 20% to 3.5%~6.1%; and the median bias decreased from 15.6 to 3.2%~5.6%. The test results indicate a significant reduction in total sample bias after matching, with the characteristics of the samples in both groups becoming more similar. The balance test results are quite favorable. By using the propensity score matching method for effect measurement and balance testing, the robustness of the empirical results is verified based on the above findings.

**Table 8:** Results of balance test.

Matching method	Pseudo-R^2^	LR chi2 (P-value)	Mean deviation.	Standard deviation.
Before matching	0.100	179.17(0.000)	20.0	15.6
Nearest Neighbour Matching	0.009	16.33(0.232)	6.1	5.6
Caliper matching	0.004	8.16(0.883)	4.1	3.4
Nuclear matching	0.003	6.15(0.941)	3.5	3.2

#### Robustness test 2: Replacement of core explanatory variables

This study conducts a robustness test by replacing the core explanatory variable "Internet Usage" with "Mobile Internet Usage" and "Computer Internet Usage" respectively. These replacement variables are derived from questionnaire responses to individually identify the impact of different internet usage modes on farmers’ adoption of fertilizer reduction and efficiency enhancement technologies. Simultaneously, samples involving both mobile and computer internet usage are excluded. Regression results are presented in Table 9. As shown in Table 9, both mobile internet usage and computer internet usage have significant positive effects on farmers’ adoption of fertilizer reduction and efficiency enhancement technologies. This further validates the effectiveness of the endogenous switching probit model.

**Table 9:** Stimation results for different internet usage modes.

Variable Names	Adopt chemical fertilizer reduction and efficiency enhancement technology
mobile internet usage	0.254** (0.163)
computer internet usage	1.368** (0.625)
Control Variables	Controlled
Observations	1295

## Conclusion

This study uses survey data from 1295 households in four prefecture-level cities in Henan Province, a typical agricultural region in China. Using an endogenous switching probit model, the study empirically investigates the impact of internet usage on farmer adoption of fertilizer reduction and efficiency enhancement technologies within a counterfactual analysis framework. The study particularly focuses on analyzing the mediating effects of expected benefits and risk perception, as well as the moderating effects of social networks.

The results indicate that. Firstly, the age, educational level, party membership, household income, land size, level of internet infrastructure, and number of mobile contacts significantly influence internet usage among farmers. This suggests that the socioeconomic factors of farmers and the level of internet infrastructure are important factors affecting their internet usage. Secondly, the age, household income, educational level, employment status, distance from the village to the city, and level of trust in the government significantly influence the adoption of fertilizer reduction and efficiency enhancement technologies among farmers. Thirdly, for farmers who already use the internet, the probability of their adopting fertilizer reduction and efficiency enhancement technologies will decrease by 28.09% if they stop using the internet. On the other hand, if farmers who do not currently use the internet for information access are able to use it, the probability of their adopting fertilizer reduction and efficiency enhancement technologies will increase by 40.67%. This further demonstrates that internet usage can effectively promote the adoption of fertilizer reduction and efficiency enhancement technologies among farmers. Fourthly, internet usage directly promotes the adoption of fertilizer reduction and efficiency enhancement technologies among farmers. It indirectly affects farmers’ adoption behavior through the mediating pathways of expected ecological benefits and expected economic benefits. Additionally, farmers can reduce their perception of risk and promote the adoption of fertilizer reduction and efficiency enhancement technologies by using the internet. Fifthly, social networks have a negative moderating effect on the relationship between internet usage and the adoption of fertilizer reduction and efficiency enhancement technologies. Internet usage has a greater promoting effect on farmers with weaker social network relationships. As the farmers’ social network relationships strengthen, the positive impact of internet usage on the adoption of fertilizer reduction and efficiency enhancement technologies gradually weakens.

## Discussion

The purpose of this study was to explore whether the use of the Internet by farmers in some areas of Henan Province, China affected their adoption of fertilizer reduction and efficiency improvement technology, and the application of Endogenous Switching probit model in the study was introduced in detail. Although we have made some valuable findings in our research, there are still shortcomings.

First of all, the research data of this study is limited to some parts of Henan Province, so the results may have certain limitations. Although we have taken measures such as sample sampling and geographical selection to minimize this limitation, the particularity of the results cannot be avoided. Therefore, we consider expanding the scope of the survey to obtain more representative and general conclusions, so as to provide more comprehensive guidance for green agricultural production. Secondly, in the application of Endogenous Switching probit model, although we chose " the number of contacts in your mobile phone " as an instrumental variable and verified its validity in the model, potential limitations and biases that may exist in the analysis of instrumental variables still cannot be excluded. Finally, this study confirmed that farmers’ use of the Internet affected their adoption of fertilizer reduction and efficiency increase technology, but its specific action path was not clear, and it was necessary to further explore the action path of the Internet on farmers’ adoption of fertilizer reduction and efficiency increase technology.
